# Machine learning models based on log odds of positive lymph nodes for predicting survival in T_1_N_+_ gastric cancer

**DOI:** 10.3389/fonc.2025.1642302

**Published:** 2026-01-09

**Authors:** Yuchen Liu, Hao Cui, Zhen Yuan, Jinghang Wang, Ruonan An, Rui Li, Jianxin Cui, Bo Wei

**Affiliations:** 1 School of Medicine, Nankai University, Tianjin, China; 2 Department of General Surgery, The First Medical Center, Chinese People’s Liberation Army General Hospital, Beijing, China

**Keywords:** early gastric cancer, lymph node metastasis, LODDS staging, machine learning, prognosis

## Abstract

**Background:**

Although early gastric cancer (EGC) is generally limited to the mucosal and submucosal layers, lymph node metastasis can still occur, which may worsen the prognosis, particularly when the number of examined lymph nodes (ELNs) is inadequate. This study introduces log odds of positive lymph nodes (LODDS) as a prognostic factor and integrates it with machine learning to improve survival predictions in T_1_N_+_ gastric cancer (GC).

**Methods:**

This retrospective study used data from the Surveillance, Epidemiology, and End Results (SEER) Program and an independent validation cohort from the Chinese People’s Liberation Army General Hospital First Medical Center. Predictive factors were selected using LASSO regression and multivariate Cox regression. Cox proportional-hazards (CoxPH), random survival forest (RSF), and XGBoost models were developed to predict overall survival (OS). Model interpretability and feature importance were evaluated using the SHapley Additive exPlanations (SHAP) method.

**Results:**

A total of 419 T_1_N_+_ GC patients from the SEER database and 193 from our institution were included in the study. LODDS staging was identified as an independent prognostic factor, demonstrating superior discriminatory power compared to N staging (C-index 0.65 vs. 0.57). Based on the Brier score, area under the ROC curve (AUC), and C-index, the RSF model outperformed both the Cox model and XGBoost model. The RSF model achieved a C-index of 0.79 in the training cohort and 0.80 in the validation cohort, indicating favorable discrimination and calibration, with Brier scores below 0.25.

**Conclusions:**

Integrating LODDS staging into the RSF model, alongside other clinical features, provides a highly accurate tool for survival prediction in T_1_N_+_ GC patients.

## 1 Introduction

Gastric cancer (GC) is an aggressive malignancy associated with poor prognosis and remains one of the leading causes of cancer-related death worldwide (1). With the widespread adoption of upper gastrointestinal endoscopic screening and advancements in diagnostic techniques, an increasing number of patients are diagnosed at earlier stages (2, 3). Early gastric cancer (EGC) is pathologically defined as tumor invasion confined to the mucosal or submucosal layers (pT_1a_, pT_1b_). Despite being categorized as early-stage disease, approximately 10–20% of EGC patients present with lymph node metastasis (T_1_N_+_) (4–6), significantly worsening their prognosis compared to those without nodal involvement (7). To better reflect the prognostic significance of the positive lymph nodes (PLNs) count, the 8th edition of the American Joint Committee on Cancer (AJCC) TNM staging system reclassified patients with T_1_N_3b_ disease from stage IIb to stage IIIb, indicating poorer survival outcomes (8). This modification underscores the ongoing debate surrounding the prognostic value of PLNs count. Furthermore, T_1_N_+_ GC has received insufficient attention, and the traditional TNM staging method relies solely on the absolute number of PLNs, which may inadequately distinguish between patients with varying prognoses (9). Thus, exploring more precise and effective lymph nodes evaluation methods is critical for achieving accurate prognostic stratification of T_1_N_+_ GC patients.

The log odds of positive lymph nodes (LODDS) represent a novel lymph node staging metric, which is defined as the natural logarithm of the ratio between the probability of being a positive lymph node and the probability of being a negative lymph node when one lymph node is retrieved (10). This novel prognostic indicator integrates both the number of PLNs and ELNs, providing a more comprehensive and accurate characterization of lymph node involvement in GC patients (11). Recent evidence has demonstrated that LODDS staging exhibit superior predictive ability compared to pathological N (pN) staging, particularly for patients undergoing extensive lymphadenectomy (12–15). Therefore, we incorporated LODDS staging into our prognostic modeling to enhance the accuracy and clinical applicability of predictions.

Currently, due to limited sample sizes, studies assessing prognostic outcomes for patients with T_1_N_+_ GC remain insufficient. Machine learning approaches have demonstrated the potential to enhance the accuracy of prognostic models by effectively managing complex clinical datasets. Therefore, we developed a machine learning-based prognostic model for patients with T_1_N_+_ GC incorporating LODDS staging, aiming to provide valuable insights for long-term survival prediction and precise risk stratification.

## 2 Material and methods

### 2.1 Patient population

**Figure 1:**
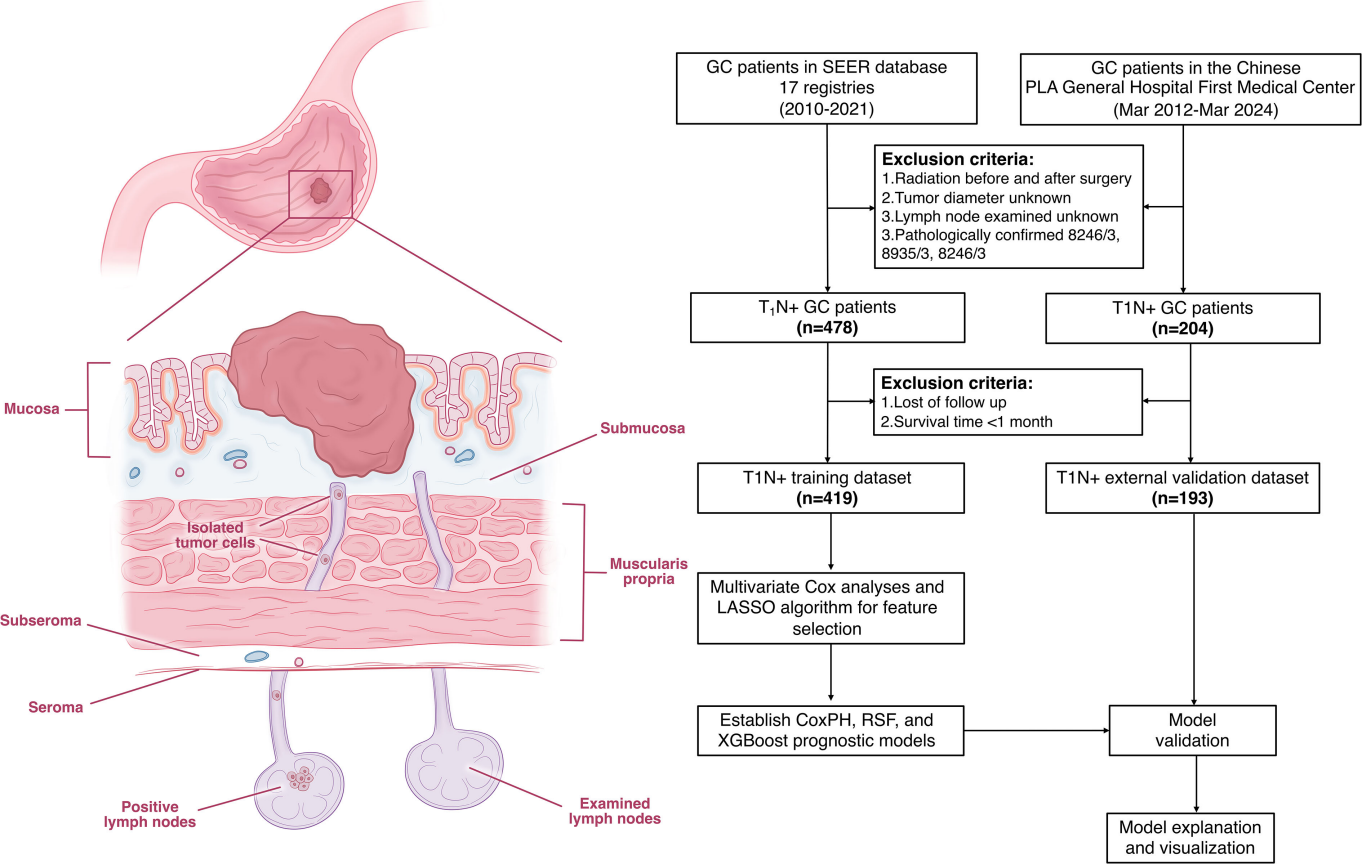
Flow chart of this study.

Data for GC patients were extracted from the Surveillance, Epidemiology, and End Results (SEER) database (SEER 17 registries, November 2023 submission, covering the years 2000–2021) of the National Cancer Institute using SEER*Stat software (version 8.4.3) (16). As the SEER database strictly maintains patient confidentiality, informed consent was waived for this retrospective analysis. Patients diagnosed with T_1_N_+_ GC between 2010 and 2021 were selected for inclusion, encompassing both those who received upfront surgery with pathological staging (pT_1_N_+_) and those who received neoadjuvant chemotherapy (NAC) followed by pathological staging (ypT_1_N_+_). Patients diagnosed with neuroendocrine carcinoma (ICD-O-3: 8246/3), stromal sarcoma (8935/3), or gastrointestinal stromal tumors (GIST) (8936/3) were excluded. Additionally, patients with missing data on tumor diameter or those who received radiotherapy or had inconsistent radiotherapy information were also excluded. After applying these inclusion and exclusion criteria, 419 eligible patients remained and were included in the final analysis (**Figure 1**).

Furthermore, data from Chinese People’s Liberation Army (PLA) General Hospital First Medical Center comprising T_1_N_+_ GC patients diagnosed between 2012 March and 2024 March were collected as a validation dataset. Employing identical inclusion and exclusion criteria, a total of 193 patients were ultimately enrolled. This study has been approved by the ethic committee of the Chinese PLA general hospital (No: S2025-234-01).

### 2.2 Study variables

Information on age at diagnosis, sex, tumor diameter, primary site, tumor differentiation, histologic type, N stage, examined lymph nodes (ELNs), positive lymph nodes (PLNs) and treatment of GC patients were collected from SEER database. Primary site was divided into proximal (ICD-O-3 code C16.0 and C16.1), middle (ICD-O-3 code C16.2), distal (ICD-O-3 code C16.4 and C16.5) and others. Histologic type was classified based on the presence or absence of signet-ring cell carcinoma (ICD-O-3 histologic code 8940). The formula of LODDS was: $LODDS=\text{log}\left(\frac{PLNs+0.5}{ELNs-PLNs+0.5}\right)$. To avoid undefined or infinite values when the number of PLNs or ELNs was zero, we added 0.5 to both the numerator and the denominator (10). The X‐tile software (3.6.1) and R package “survminer” were used to determine the optimal cutoff values for the three continuous variables of age, tumor diameter, and LODDS staging according to overall survival (OS). Tumor diameter was classified as<10 and >10 mm; LODDS stage was classified as LODDS1 (<−0.9), LODDS2 (−0.9 to −0.4), and LODDS3 (>= −0.4).

### 2.3 Follow-up

The primary endpoint was overall survival (OS), defined as the interval from the date of diagnosis to the date of death, the date last known to be alive, or the study cut-off date. Patients from PLA General Hospital follow-up data were collected by trained assistants after patient discharge and reviewed by a senior attending physician prior to analysis. Patients received postoperative follow-ups every 3–6 months for the first two years, every 6–12 months from the third through the fifth year, and annually thereafter, according to the 2023 Chinese Society of Clinical Oncology (CSCO) guidelines for gastric cancer management (17). The median follow-up time for the SEER dataset was 80 months (95% confidence interval (CI): 66–111 months) and external validation dataset was 71 months (95% CI: 65–80 months).

### 2.4 Model development and evaluation

Multivariate Cox regression analyses and the least absolute shrinkage and selection operator (LASSO) algorithm were conducted using clinical characteristics derived from the SEER database. The LASSO regression was conducted using the R package “glmnet”, which applies L1 regularization to shrink regression coefficients and select relevant variables while avoiding overfitting. Variables identified as statistically significant in Cox analyses or considered clinically relevant, including age at diagnosis, primary site, LODDS staging, N staging, treatment modality, and tumor diameter, were integrated into machine learning models to predict 1-, 3-, and 5-year OS for T_1_N_+_ gastric cancer (GC) patients.

Machine learning models were constructed using the mlr3proba framework in R (18), which provides a standardized environment for model training, hyperparameter tuning, and performance evaluation under unified resampling strategies. Two machine learning algorithms: Random Survival Forest (RSF) and eXtreme Gradient Boosting (XGBoost) were implemented to predict OS. Model hyperparameters were optimized through a grid search combined with 10-fold cross-validation on the training cohort to maximize the mean concordance index (C-index). Following grid-search optimization, the RSF model’s optimal configuration consisted of 500 estimators, with both the minimum samples required to split an internal node and the minimum samples per terminal node set to 10. For the XGBoost model, the optimal parameters were 300 boosting rounds, a learning rate of 0.05, a maximum tree depth of 4, a minimum child weight of 2 and a subsample ratio of 0.8. All models were trained with fixed random seeds to ensure reproducibility.

To evaluate predictive performance, receiver operating characteristic (ROC) curves and the corresponding area under the curve (AUC) values were compared among the CoxPH, RSF and XGBoost models. Additionally, decision curve analysis (DCA) and Brier scores were employed to assess the clinical utility, precision, and accuracy of these predictive models. To further validate the RSF prognostic model, data from an independent cohort of 193 patients diagnosed with T_1_N_+_ GC at our institution were collected and analyzed.

Interpretability of the prognostic model was crucial for facilitating clinical decision-making, enabling physicians to transparently comprehend the factors influencing postoperative outcomes. Furthermore, the SHapley Additive exPlanations (SHAP) approach, a game-theoretic method, was utilized to illustrate the contribution of individual variables to model predictions, enhancing clinical interpretability (19). SHAP values were computed and plotted using the R package “shapviz”.

### 2.5 Statistical analysis

Differences in demographic and clinical characteristics between the training and validation cohorts were evaluated using the “tableone” R package. The Wilcoxon test for continuous variables, and either the χ² test or Fisher’s exact test for categorical variables. To investigate the associations between clinical-pathological factors and OS among T_1_N_+_ GC patients, univariate Cox analyses were performed using the “survival” R package. Variables with p< 0.05 in the univariate analyses were subsequently included in multivariate Cox regression analyses to further evaluate mortality risk and identify independent prognostic factors.

To examine the prognostic impact of NAC in patients with T_1_N_+_ GC, patients receiving NAC were matched with those who did not receive NAC using a 1:1 propensity score matching (PSM) approach implemented in the “MatchIt” R package (20, 21). Propensity scores were estimated using a multivariable logistic regression model that included relevant covariates from the SEER database. Two-tailed p-values of less than 0.05 were considered statistically significant. All statistical analyses were performed using R software (Version 4.2.3, Vienna, Austria).

## 3 Results

### 3.1 The characteristics of patients

Overall, 419 patients with T_1_N_+_ GC diagnosed between 2010 and 2021 were identified from the SEER database and used as the training dataset. Additionally, 193 patients from Chinese PLA General Hospital First Medical Center were included as a validation dataset. **Table 1** summarizes the demographic and clinical characteristics of these two patient cohorts. Patients in the validation dataset had a lower mean age (58.3 vs. 67.9 years, p<0.001) and a higher proportion of patients with N2 stage (45.1%), whereas patients in the training cohort predominantly presented with stage N1 (70.9%). Furthermore, the proportion of patients who received NAC was lower in the validation dataset compared to the training dataset (5.2% vs. 27.0%, p<0.001).

**Table 1:** Demographic and clinicopathological characteristics of training dataset and validation dataset.

Variable	Training dataset	Validation dataset	*P* value
	(n=419)	(n=193)	
Age (mean (SD))	67.91 (13.04)	58.27 (11.40)	<0.001
Sex (%)			0.515
Female	169 (40.3)	68 (35.2)	
Male	250 (59.7)	125 (64.8)	
N stage (%)			<0.001
N1	297 (70.9)	77 (39.9)	
N2	87 (20.8)	87 (45.1)	
N3	35 (8.4)	29 (15.0)	
LODDS stage (%)			0.201
LODDS1	123 (29.4)	71 (36.8)	
LODDS2	232 (55.4)	98 (50.8)	
LODDS3	64 (15.3)	24 (12.4)	
Tumor diameter (%)			0.005
> 10mm	340 (81.1)	173 (89.6)	
≤ 10 mm	79 (18.9)	20 (10.4)	
Primary site (%)			<0.001
Distal	122 (29.1)	125 (64.8)	
Proximal	127 (30.3)	23 (11.9)	
Middle	61 (14.6)	36 (18.7)	
Nos	109 (26.0)	9 (4.7)	
Tumor differentiation (%)			<0.001
Well	16 (3.8)	9 (4.7)	
Moderate	68 (16.2)	55 (28.5)	
Poor/undifferentiated	114 (27.2)	129 (66.8)	
Unknown	221 (52.7)	0 (0.0)	
Signet-ring carcinoma (%)			<0.001
Absence	367 (87.6)	114 (59.1)	
Presence	52 (12.4)	79 (40.9)	
Treatment (%)			<0.001
NAC	113 (27.0)	10 (5.2)	
Non-NAC	306 (73.0)	183 (94.8)	

LODDS, log odds of positive lymph nodes; NAC, neoadjuvant chemotherapy.

### 3.2 Comparison between LODDS and N staging for prognosis

**Figure 2:**
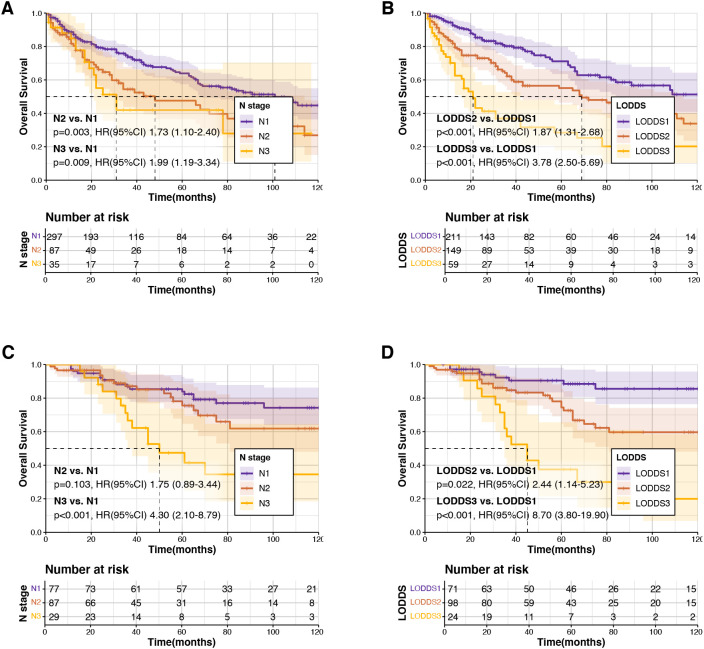
Kaplan–Meier survival analysis comparing N staging and log odds of positive lymph nodes (LODDS) staging in training and validation datasets. **(A, B)** N staging and LODDS staging for overall survival (OS) in the training dataset. **(C, D)** N staging and LODDS staging for overall survival (OS) in the validation dataset.

In terms of the N staging system, a significant difference in prognosis was observed between patients classified as N_1_ compared to those classified as N_2_ and N_3_ combined (p<0.001, hazard ratio (HR)=1.80, 95% CI: 1.31–2.78). However, there was no statistically significant prognostic difference between N_2_ and N_3_ stages (p=0.6, HR = 1.16, 95% CI: 0.66–2.05). Conversely, the LODDS staging system demonstrated superior prognostic discrimination among patient groups (**Figure 2**). Furthermore, LODDS staging demonstrated superior predictive accuracy compared to N staging, as indicated by higher concordance indices, with C-indices of 0.65 versus 0.57 in the training dataset and 0.67 versus 0.60 in the validation dataset, respectively. ROC analyses for predicting 1-, 3-, and 5-year OS further confirmed these findings (**Supplementary Figure S1**).

Multivariate Cox regression analyses revealed that LODDS staging was significantly associated with OS (p<0.05), while N_3_ stage was not identified as an independent prognostic factor (p>0.05). Additionally, older age, and tumor diameter emerged as independent prognostic factors significantly influencing OS (**Table 2**).

**Table 2:** Univariate and multivariate Cox analysis of characteristics extracted from SEER database.

Variable	Univariate OS		Multivariate OS	
	P value	HR (95%CI)	P value	HR (95%CI)
**Age**	**<0.001**	1.03 (1.02-1.05)	**0.003**	1.02 (1.01-1.04)
Sex				
Female	Ref.			
Male	0.053	1.38 (0.99-1.92)		
N stage				
N1	Ref.		Ref.	
N2	**0.003**	1.73 (1.10-2.40)	**0.03**	1.56 (1.05-2.32)
N3	**0.009**	1.99 (1.19-3.34)	0.47	1.25 (0.68-2.30)
LODDS stage				
LODDS1	Ref.		Ref.	
LODDS2	**<0.001**	1.87 (1.31-2.68)	**0.03**	1.57 (1.05-2.33)
LODDS3	**<0.001**	3.78 (2.50-5.69)	**<0.001**	2.95 (1.83-4.78)
Primary site				
Distal	Ref.			
Middle	0.07	0.59 (0.34-1.05)		
Proximal	0.1	1.37 (0.93-2.03)		
Nos	0.79	0.94 (0.62-1.44)		
Tumor size				
> 10 mm	Ref.		Ref.	
≤ 10 mm	**0.002**	0.47 (0.30-0.75)	**0.03**	0.59 (0.37-0.95)
Tumor differentiation				
Well	Ref.			
Moderate	0.08	2.55 (0.91-7.13)		
Poor/undifferentiated	0.1	2.48 (0.89-6.85)		
Unknown	0.42	1.54 (0.54-4.41)		
Signet-ring carcinoma				
Absence	Ref.		Ref.	
Presence	**0.01**	0.51 (0.30-0.87)	0.07	0.61 (0.35-1.05)
Treatment				
NAC	Ref.			
Non-NAC	0.098	1.41 (0.94-2.13)		

CI, confidence interval; HR, hazard ratio; LODDS, log odds of positive lymph nodes; NAC, neoadjuvant chemotherapy; OS, overall survival.Variables with statistically significant associations (p < 0.05) are presented in bold.

### 3.3 Features selection

**Figure 3:**
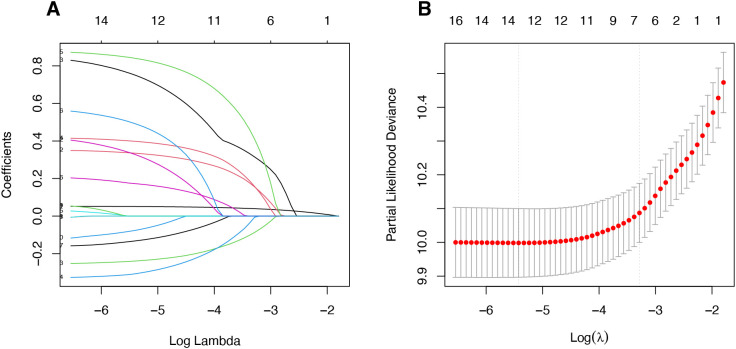
Results of the least absolute shrinkage and selection operator (LASSO) regression analysis for the prediction models: **(A)** coefficient profiles plotted against log(λ); **(B)** ten-fold cross-validation demonstrating the optimal λ value.

The LASSO algorithm and Cox regression analyses were employed to select the variables in the study. Two variables, the tumor differentiation and signet-ring carcinoma were excluded, and the remaining 6 variables were included in the construction of the RSF model. These remaining variables included age, tumor diameter, primary site, N staging and LODDS staging. The procedures for selecting variables are shown in **Figure 3**.

After variables selecting, to ensure that there was no collinearity among the variables, we utilized Spearman correlation analysis. The correlation analysis revealed no strong correlations among the included variables (|r|< 0.5 for all pairs, and most p > 0.05). This indicates the absence of significant multicollinearity, suggesting that these variables can be simultaneously included in subsequent model construction (**Supplementary Figure S2**).

### 3.4 Model development and performance comparison

**Figure 4:**
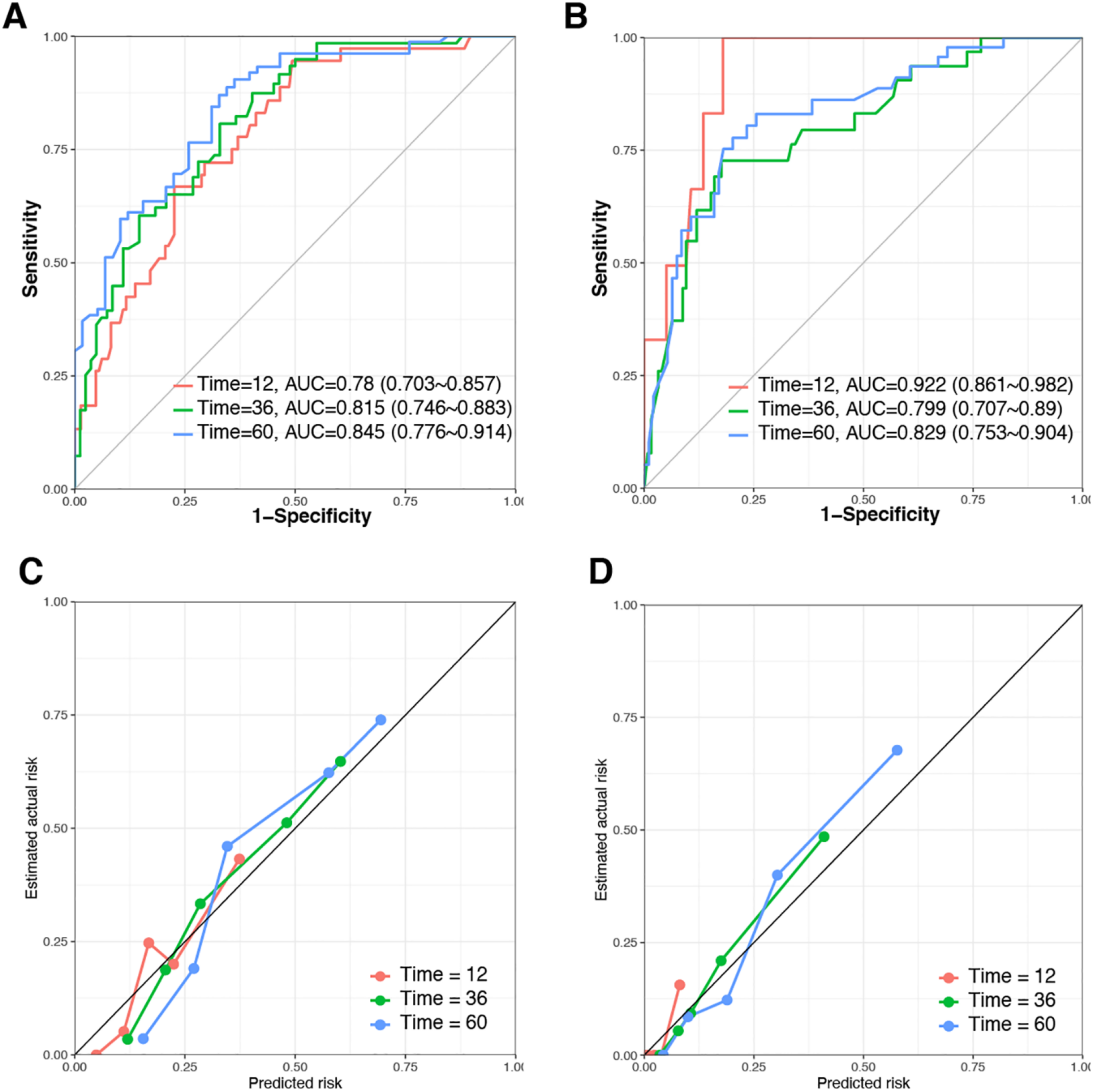
Evaluation of the Random Survival Forest (RSF) model in the training and validation datasets. **(A, B)** Time‐dependent AUC and receiver operating characteristic (ROC) curves in the training and validation datasets. **(C, D)** Calibration curves of RSF model in the training and validation dataset.

**Figure 5:**
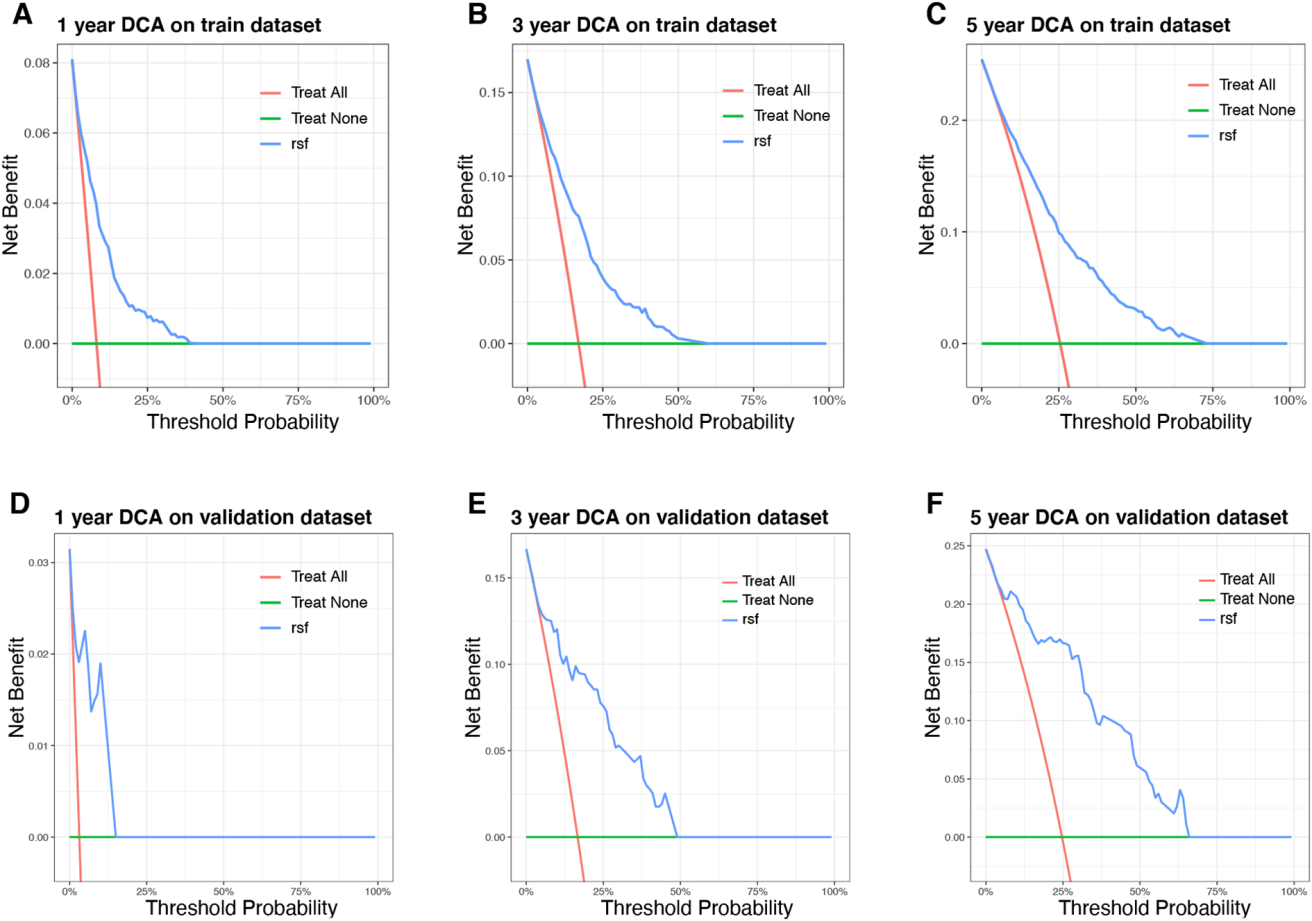
The decision curve analysis (DCA) of the Random Survival Forest (RSF) model. **(A–C)** The 1-, 3-, 5-year DCA of the RSF model on training dataset. **(D–F)** The 1-, 3-, 5-year DCA of the RSF model on validation dataset.

In this study, the developed prognostic models were validated using a dataset to assess the predictive performance. Among these models, the RSF algorithm demonstrated superior predictive accuracy compared to the CoxPH and XGBoost models. Specifically, the RSF model achieved the highest C-index of 0.785 and demonstrated the highest AUC for predicting 1-, 3-, and 5-year OS in both the training and validation datasets (**Table 3**; **Figures 4A, B**). All evaluated models exhibited Brier scores below 0.25, indicating robust calibration, with the RSF model notably displaying the lowest scores. Calibration curves further validated the excellent predictive accuracy of the RSF model (**Figures 4C, D**). Additionally, DCA showed substantial clinical net benefits of the RSF model for survival prediction at 1-, 3-, and 5-year OS (**Figure 5**).

**Table 3:** Models’ evaluation of training dataset and validation dataset.

Model	C-index	AUC			Brier score		
		1-year	3-year	5-year	1-year	3-year	5-year
CoxPH model							
Training cohort	0.718	0.745	0.73	0.746	0.134	0.2	0.205
Validation cohort	0.752	0.775	0.788	0.812	0.032	0.115	0.138
RSF model							
Training cohort	0.785	0.780	0.815	0.845	0.125	0.174	0.179
Validation cohort	0.801	0.922	0.799	0.829	0.029	0.116	0.134
XGBoost model							
Training cohort	0.757	0.766	0.775	0.787	0.129	0.189	0.194
Validation cohort	0.741	0.718	0.76	0.814	0.031	0.112	0.139

AUC, area under the ROC curve; CoxPH: cox proportional hazards; RSF, random survival forest; XGBoost, extreme gradient boosting.

### 3.5 Model explanation

**Figure 6:**
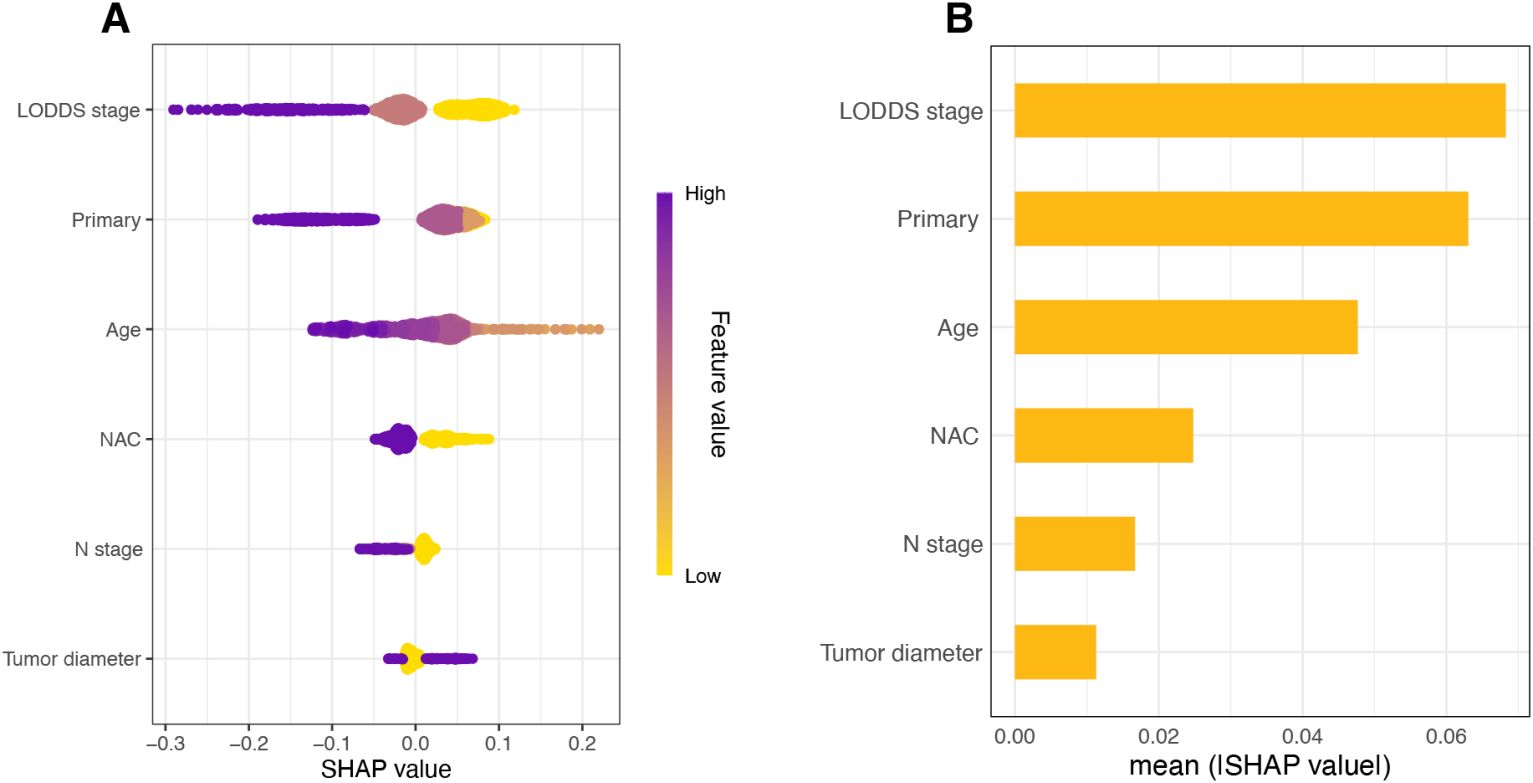
Global model explanation by the SHapley Additive exPlanations (SHAP) method. **(A)** SHAP summary dot plot. **(B)** SHAP summary bar plot.

The RSF model demonstrated optimal performance on both the training and validation datasets. To further clarify the contribution of each predictor variable and enhance the interpretability of the RSF model, we assessed feature importance using SHAP. The SHAP summary plots display the predictors ranked in descending order according to their average SHAP values, reflecting their relative contributions to the model predictions (**Figure 6**). Among these variables, LODDS staging emerged as the most influential factor (0.0683), substantially surpassing the importance of N staging (0.0167).

### 3.6 Impact of NAC on ypT_1_N_+_ GC prognosis

**Figure 7:**
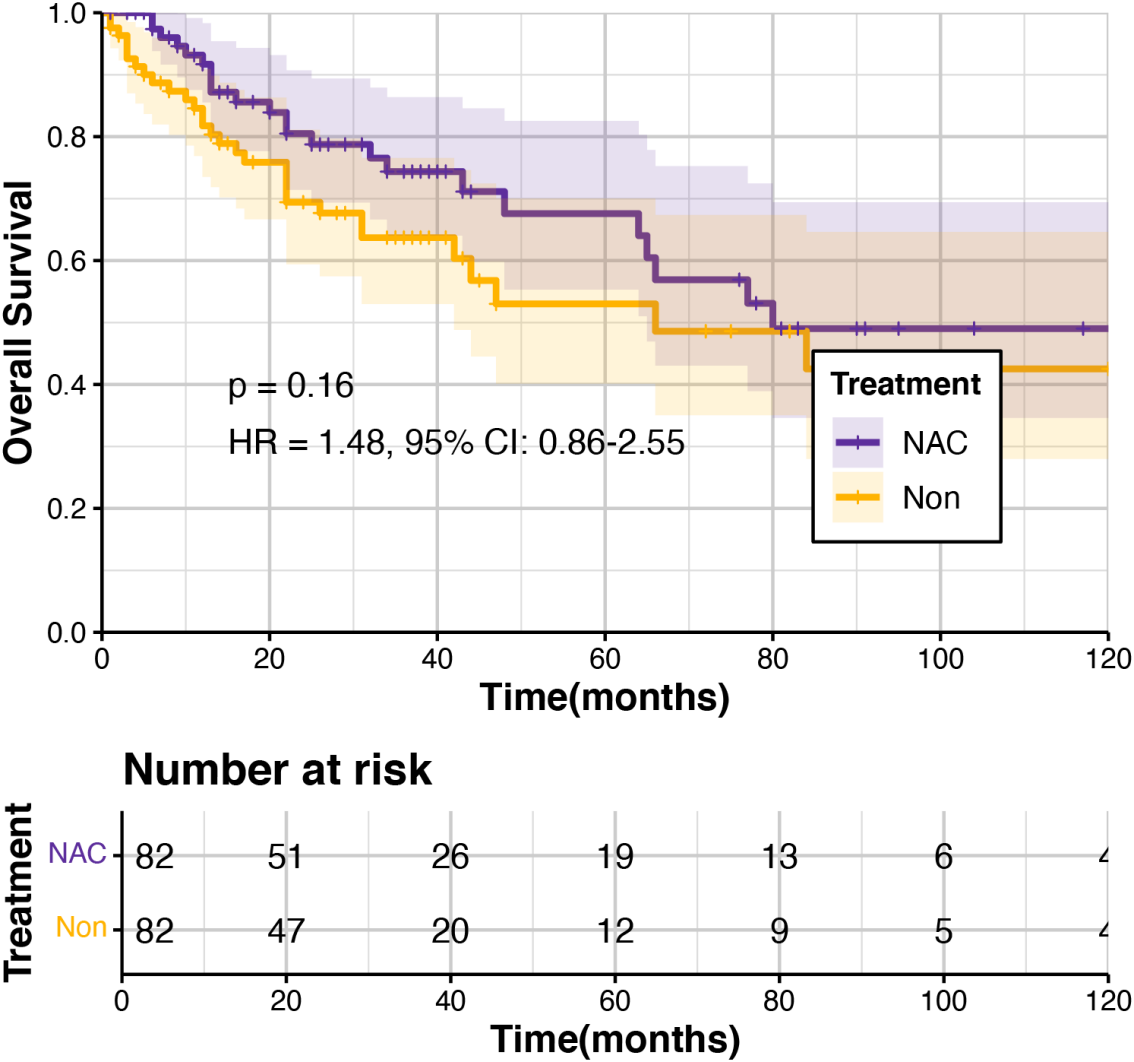
Kaplan-Meier plot of pT_1_N_+_ patients who underwent upfront surgery and ypT_1_N_+_ patients treated with neoadjuvant chemotherapy (NAC).

Additionally, we compared the prognosis of ypT_1_N_+_ GC patients who underwent perioperative chemotherapy with pT_1_N_+_ patients who did not receive NAC using data from the SEER database. Considering the imbalance in baseline characteristics between these two groups, we conducted a 1:1 propensity score matching (PSM) analysis to ensure comparability (**Supplementary Table S1**). After matching, our results indicated no significant difference in OS between pT_1_N_+_ patients who underwent upfront surgery alone and ypT_1_N_+_ patients treated with NAC (p = 0.159, HR = 1.48, 95% CI: 0.86–2.55) (**Figure 7**).

## 4 Discussion

Early-stage GC generally exhibits a favorable prognosis, and satisfactory survival outcomes can typically be achieved with surgery alone (22). However, T_1_ GC patients with lymph node metastasis (T_1_N_+_) tend to have significantly worse prognoses and have not received sufficient attention (23). To the best of our knowledge, this is the first study to develop an artificial intelligence (AI)-based predictive model specifically designed to evaluate long-term outcomes in patients with T_1_N_+_ GC. Furthermore, the RSF model maintained good discrimination and calibration in the independent validation cohort, supporting its robustness and potential clinical utility.

Although the AJCC TNM staging system is widely used in clinical practice and provides valuable prognostic information, it has certain limitations, particularly in T_1_N_+_ GC patients. One key limitation is that N staging only considers PLNs and does not account for ELNs, which have also been shown to influence prognosis (24). Our results further demonstrate that N staging does not adequately distinguish between patient prognoses, this limitation also highlighted in the study by Que et al. (25). To overcome this limitation, we developed a predictive model based on LODDS staging, which incorporates both PLNs and ELNs into the calculation. The results indicated that LODDS staging outperforms N staging in predicting survival outcomes for T_1_N_+_ GC patients and identifying high-risk populations. A systematic review by Li et al. confirmed that LODDS is strongly correlated with GC patient prognosis and provides a more accurate prediction of survival than earlier methods. Additionally, the utility of LODDS staging has been validated in studies across various tumor types (26–29).

In the SEER cohort, discrepancies were observed between N staging and LODDS staging, likely attributable to inaccuracies in staging caused by an insufficient number of ELNs. Previous studies and AJCC guidelines recommend examining at least 16 lymph nodes to ensure accurate staging (8, 30). However, there remains ongoing debate regarding whether collecting a greater number of lymph nodes translates into survival benefits. While extensive lymph node dissection can enhance staging precision and guide adjuvant treatment decisions, it may also increase postoperative complications and morbidity. Therefore, determining an optimal range of ELNs is essential for balancing accurate staging with patient safety (31).

This prognostic model, based on the RSF algorithm, was constructed to predict both short-term and long-term survival outcomes in patients with T_1_N_+_ GC. The RSF algorithm, first introduced in 2008, has since become a widely accepted method for survival analysis and prognosis prediction (32). Compared with previous Cox regression analysis, RSF algorithm demonstrates superior performance, particularly when handling high-dimensional data (33). In the current study, our RSF model exhibited enhanced calibration and discrimination in predicting 1-, 3-, and 5-year OS for T_1_N_+_ GC patients in both the SEER-based training dataset and the validation dataset from our institution, outperforming the Cox regression and XGBoost models. Thus, the RSF model shows considerable promise in enhancing the accuracy and reliability of individualized prognostic predictions.

To overcome the “black-box” challenge of machine learning models, we employed the SHAP method to interpret our RSF model and visualize the contribution of individual predictors (19). The results indicated that LODDS staging was the most important factor, reinforcing its critical prognostic value. Primary tumor site and age at diagnosis ranked second and third, respectively, aligning with findings from previous research. In addition, multivariate Cox analysis confirmed that age and proximal GC serve as independent prognostic factors. Our findings indicate a poorer prognosis in older patients, consistent with observations by Choi et al. (34), which may be due to increased comorbidities associated with treatment and nutritional complications in this population (35, 36). Furthermore, the poor prognosis associated with proximal GC has been widely reported, which may be attributed by its distinct morphology, clinical behavior, and therapeutic responses—suggesting that proximal GC constitutes a relatively independent disease subset (37, 38).

NAC is increasingly utilized in the treatment of GC. However, controversy persists regarding prognostic differences between ypT_1_ GC patients who underwent NAC and pT_1_ GC patients who received upfront surgery (39, 40). In this study, we compared the prognoses of these two patient populations and found that the prognosis of patients classified as ypT_1_ following NAC was comparable to that of patients initially diagnosed at pathological stage pT_1_. These findings align with previous studies and further support the perspective that ypT_1_ GC patients generally have favorable clinical outcomes. This result proves the important role of NAC in the treatment of GC patients.

Our study has several limitations that should be acknowledged. First, as a retrospective analysis, it is inherently subject to missing data and selection bias. Second, due to the limited information available in the SEER database, potentially important prognostic variables such as tumor biomarkers (e.g., CEA and CA72-4) were not included in the analysis (41–43). Moreover, detailed treatment-related information, including NAC regimens and treatment durations, were not provided in the SEER database. In addition, some patients had relatively short follow-up durations, although the median follow-up time was adequate for survival estimation. Continued follow-up and data collection from additional centers are warranted to further strengthen the model’s robustness and generalizability. Lastly, given the retrospective nature of this study, prospective multicenter clinical studies are needed to further validate and confirm the clinical applicability of our prognostic model.

In conclusion, this study analyzed the clinical characteristics of patients with T_1_N_+_ GC and developed three prognostic models to predict survival outcomes. Among these, the RSF model demonstrated the best predictive performance and validated in an external cohort. Additionally, we identified key prognostic factors for T_1_N_+_ GC. Our results indicate that patients diagnosed as ypT_1_ following neoadjuvant chemotherapy have comparable survival outcomes to those initially staged as pT_1_.

## Data Availability

The raw data supporting the conclusions of this article will be made available by the authors, without undue reservation.

## Supplementary material

Supplementary Figure 1 The correlation heat map of the parameters. Red indicates a positive correlation and blue indicates a negative correlation.

Supplementary Figure 2 Receiver operating characteristic (ROC)of N staging and log odds of positive lymph nodes (LODDS) staging. “ns” indicates not significant; *p* < 0.05 (*), p< 0.01 (**), and p< 0.001 (***).
