# Supplementary material for: Machine learning models based on log odds of positive lymph nodes for predicting survival in T1N+ gastric cancer
Source: Front Oncol. 2026 Jan 9;15:1642302. doi: 10.3389/fonc.2025.1642302 (PMC12827109; doi:10.3389/fonc.2025.1642302)
Supplement: Supplementary Figure 1 — The correlation heat map of the parameters. Red indicates a positive correlation and blue indicates a negative correlation. [file DataSheet1.docx]

Supplementary Material

# Supplementary Figures and Tables

## Supplementary Figures


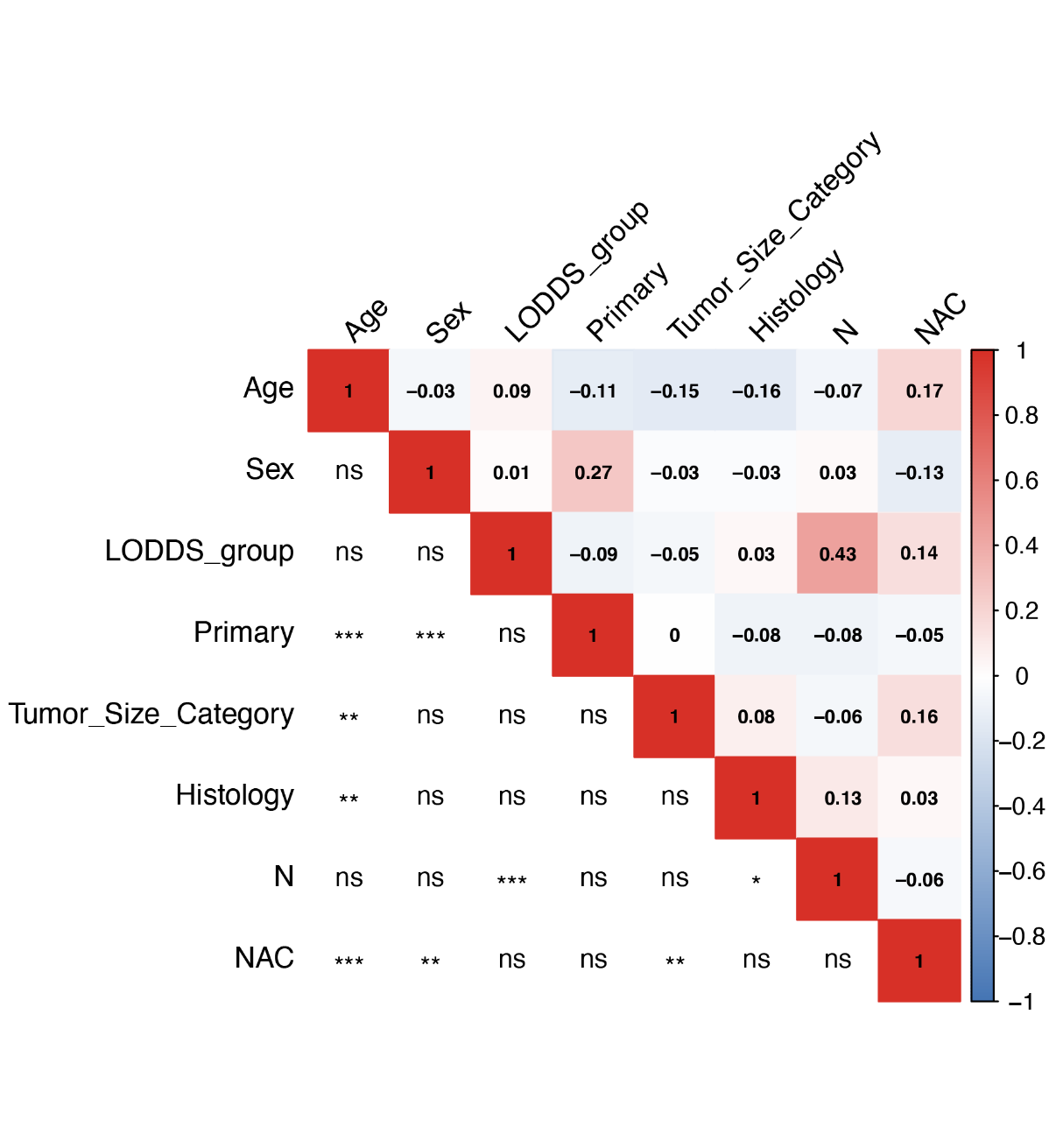


**Supplementary Figure 1.** The correlation heat map of the parameters. Red indicates a positive correlation and blue indicates a negative correlation

**Supplementary Figure 2.** ROC of N staging and LODDS staging.

## Supplementary Table

**Supplementary Table 1**. Demographic and clinicopathological characteristics of patients who received NAC (ypT1N+) and those who underwent upfront surgery (pT1N+).

| **Variable** | **NAC (ypT1N+)** | **Non-NAC**  **(pT1N+)** | ***p*** | **NAC (ypT1N+)** | **Non-NAC**  **(pT1N+)** | ***p*** |
| --- | --- | --- | --- | --- | --- | --- |
|  | (n=113) | (n=306) |  | (n=82) | (n=82) |  |
| **Age (mean (SD))** | 64.30 (12.02) | 69.25 (13.16) | 0.001 | 65.79 (11.92) | 66.02 (15.88) | 0.916 |
| **Sex (%)** |  |  | 0.013 |  |  | 0.414 |
| Female | 34 (30.1) | 135 (44.1) |  | 26 (31.7) | 32 (39.0) |  |
| Male | 79 (69.9) | 171 (55.9) |  | 56 (68.3) | 50 (61.0) |  |
| **N stage (%)** |  |  | 0.365 |  |  | 0.642 |
| N1 | 77 (68.1) | 220 (71.9) |  | 54 (65.9) | 58 (70.7) |  |
| N2 | 23 (20.4) | 64 (20.9) |  | 21 (25.6) | 16 (19.5) |  |
| N3 | 13 (11.5) | 22 (7.2) |  | 7 (8.5) | 8 (9.8) |  |
| **LODDS stage (%)** |  |  | <0.001 |  |  | 0.861 |
| LODDS1 | 59 (52.2) | 64 (20.9) |  | 36 (43.9) | 37 (45.1) |  |
| LODDS2 | 33 (29.2) | 199 (65.0) |  | 33 (40.2) | 30 (36.6) |  |
| LODDS3 | 21 (18.6) | 43 (14.1) |  | 13 (15.9) | 15 (18.3) |  |
| **Tumor diameter (%)** |  |  | 0.002 |  |  | 1 |
| > 10 mm | 103 (91.2) | 237 (77.5) |  | 72 (87.8) | 72 (87.8) |  |
| ≤ 10 mm | 10 (8.8) | 69 (22.5) |  | 10 (12.2) | 10 (12.2) |  |
| **Primary site (%)** |  |  | 0.314 |  |  | 0.546 |
| Distal | 28 (24.8) | 94 (30.7) |  | 22 (26.8) | 25 (30.5) |  |
| Proximal | 14 (12.4) | 47 (15.4) |  | 14 (17.1) | 11 (13.4) |  |
| Middle | 36 (31.9) | 73 (23.9) |  | 24 (29.3) | 18 (22.0) |  |
| Nos | 35 (31.0) | 92 (30.1) |  | 22 (26.8) | 28 (34.1) |  |
| **Tumor differentiation (%)** |  |  | <0.001 |  |  | 0.963 |
| Well | 0 (0.0) | 16 (5.2) |  | -- | -- |  |
| Moderate | 7 (6.2) | 61 (19.9) |  | 7 (8.5) | 8 (9.8) |  |
| Poor/undifferentiated | 19 (16.8) | 95 (31.0) |  | 19 (23.2) | 19 (23.2) |  |
| Unknown | 87 (77.0) | 134 (43.8) |  | 56 (68.3) | 55 (67.1) |  |
| **Signet-ring carcinoma (%)** |  |  | 0.611 |  |  | 1 |
| No | 101 (89.4) | 266 (86.9) |  | 73 (89.0) | 74 (90.2) |  |
| Yes | 12 (10.6) | 40 (13.1) |  | 9 (11.0) | 8 (9.8) |  |
